# Outcomes after arthroscopic revision surgery for anterior cruciate ligament injuries

**DOI:** 10.1080/17453674.2021.1897744

**Published:** 2021-03-19

**Authors:** Alexei V Yumashev, Tatyana V Baltina, Dmitrii V Babaskin

**Affiliations:** a Department of Prosthetic Dentistry, Sechenov First Moscow State Medical University (Sechenov University) , Moscow ;; b Institute of Fundamental Medicine and Biology, Kazan Federal University , Kazan ;; c Department of Pharmacy, Sechenov First Moscow State Medical University (Sechenov University) , Moscow , Russian Federation

## Abstract

Background and purpose — The frequency of primary anterior cruciate ligament (ACL) reconstruction is increasing resulting in more ACL revision surgeries. Therefore, we assessed survival rates of 2 different grafts for ACL revision surgery at 1- and 5-year follow-ups, as well as physical activity levels of patients after revision surgery.

Patients and methods — This is a retrospective cohort study involving 218 patients (176 males) who had revision surgery for anterior cruciate ligament injuries between 2008 and 2017 at the Clinic of Traumatology, Orthopedics and Joint Pathology Clinic (I.M. Sechenov First Moscow State Medical University). A comparison group involved 189 patients with only primary surgery. Surgical interventions were performed according to the standard procedure using bone–patellar tendon–bone (BTB) and semitendinosus/gracilis (ST/G) autografts. The results of revision surgery were assessed at 1- and 5-year follow-ups by using the Lysholm and International Knee Documentation Committee scores.

Results — Malpositioned bone tunnels were found in 87/218 patients (40%). At 1 and 5 years postoperatively, the revision BTB group had significantly better results in terms of IKDC and Lysholm scores than the revision ST/G group (p = 0.03, Mann–Whitney U-test), and these results were comparable to those in the comparison group. Graft survival after revision was lower than after the primary operation. However, the survival rate of 80% is quite high and is consistent with previous findings. There were no statistically reliable differences in survival between ST/G and BTB autografts.

Interpretation — The graft choice for revision ACL surgery should be decided upon before surgery based on, among other things, the state of bone tunnels, in particular their position and degree of bone resorption. Tunnel widening that exceeds 14 mm (osteolysis) would require 2-stage surgery using a BTB autograft with bone plugs because it is larger than the ST/G autograft.

Anterior cruciate ligament (ACL) repair has a steadily increasing success rate, varying between 75% and 95% (Lee et al. 2012, Shin et al. 2014). However, the need for revision surgery increases as the number of primary ACL reconstructions grows. Subjective and objective joint instability indicates graft failure and is the major indication for revision (Mohtadi et al. 2011, Magnussen et al. 2012).

Revision surgery is more complex than primary reconstruction, since initially improper positioning of the bone tunnels complicates the creation of new tunnels and can entail incorrect graft choice (Shafizadeh et al. 2014). Thus, the success rate of surgery is also dependent on graft-fixation choice.

Optimal graft choice and technique for revision ACL surgery remain an open debate. There is insufficient evidence for differences in long-term functional outcome between BTB (bone–patellar tendon–bone) and ST/G (semitendinosus/gracilis) grafts (Mohtadi et al. 2011). Despite preferences towards autografts (Ferretti et al. 2002, Gladilina al. 2018), some authors recommend using allografts as the least traumatic option (Bull et al. 2002). However, the choice of graft for revision surgery has been reported not to affect long-term outcomes (Ruiz et al. 2002).

The interval between revision surgery and injury is another matter of debate. Early ACL revision may have a higher success rate; late interventions may result in degenerative joint disease (Shin et al. 2014). Degenerative changes in the cartilage and meniscal injuries occur with greater frequency during revision than during the primary ACL reconstruction (Stergios et al. 2012).

This study aims to determine which graft is best for ACL revision surgery by looking at the survival rate of BTB and ST/G autografts and the physical activity levels of patients in the 1- and 5-year postoperative period. Knee outcomes are projected to be better after arthroscopic revision treatment with an autograft, but the autograft survival rate at revision may be lower than after primary surgery. We also investigated the role of bone tunnel malposition in the re-emergence of knee instability.

## Patients and methods

### Study design

This study includes 218 patients who underwent ACL revision surgery between 2008 and 2017 at the Traumatology, Orthopedics and Joint Pathology Clinic, I.M. Sechenov First Moscow State Medical University (study group). There were 176 men and 42 women aged 19 to 36 years. The inclusion criterion was a recurrent knee instability after primary ACL repair, reported to occur less than a year before complaint. The reported injury mechanisms of recurrent knee instability were sports activities (71%) and household accidents (17%); 12% of patients denied injury, reporting that knee instability persisted after the primary ACL surgery. The primary ACL surgery was by an autogenous BTB autograft (44%), an ST/G autograft (38%), or a synthetic graft (18%).

The comparison or control group involved 180 patients, including 135 males and 45 females aged 19–42 years, who underwent primary ACL surgeries within the same time period as the study group. In the control group, 68% of patients had suffered an ACL rupture due to sports injury and 32% of patients had ruptures due to household injuries. BTB autografts were used in 52% of patients, while the ST/G hamstring autografts were used to treat ACL injuries in 48% of patients.

All revision surgeries were done by the same surgical team at the Traumatology, Orthopedics and Joint Pathology Clinic. Patients in the study group underwent primary surgery at different clinics, and patients in the control group were primary operated at Traumatology, Orthopedics and Joint Pathology Clinic. The surgical team performing primary surgery on patients in the control group was the same team that did revision surgeries among patients in the study group. For both the control and study groups, the exclusion criteria were patients with damage to the meniscus, and cartilage defects.

### Procedures

To diagnose the ACL injury, 3 common tests were applied: the Lachman test, the anterior drawer test, and the pivot-shift test. Knee arthrometry was performed using a KT-1000 knee arthrometer (MEDmetric Corp, San Diego, CA, USA). Results from the Lachman and anterior drawer tests were then compared with the KT-1000 measurements. The study included patients who had positive Lachman tests, 2+ anterior drawer tests or greater, more than 3 mm of displacement between healthy and injured joints on KT 1000 testing, and grade 2 pivot shift tests or greater (noticeable displacement, rough slide with a click).

All revision patients underwent frontal and lateral radiography in the supine position to depict the size and position of the bone tunnels. In some cases, patients were assigned to receive computed tomography (CT) for a more accurate evaluation, which was crucial for good preoperative planning. The preoperative planning also involved three-dimensional (3D) reconstruction of the knee joint using CT images. Patients with no contraindications also underwent MRI scanning with a view to evaluating the strength and type of graft fixation.

The tibial bone tunnel was classified as positioned correctly if, on the frontal radiograph, it formed an angle between 60°and 65° with respect to the medial joint line of the tibia; and, on the lateral radiograph, the tunnel was posterior and oriented parallel to the Blumensaat line. The femoral bone tunnel was classified as positioned correctly if it met the following criteria: in the ‘over-the-top position’; the tunnel was located 2 mm ventral to the posterior cortical layer of the femur.

### Revision arthroscopic knee procedure

The choice of treatment procedure for the management of bone tunnels depended on the position thereof after primary surgery. When the previous tunnel did not interfere with new tunnel placement, it was left intact. In cases of tunnel overlap the hardware removal was mandatory, and the previous tunnels were filled with osteoplastic material. Bone defects that remain after hardware removal can be substantial, as in the case of osteolysis, and require filling with a bone plug.

In cases of massive osteolysis (tunnel diameter of ≥ 14 mm) a 2-stage approach was used. The second stage of the revision took place after the 4–6-month mark when the previous tunnels filled with osteoplastic material showed the radiographic signs of consolidation. The 1-stage approach was used in other cases.

The graft was chosen before surgery. The graft fixation type was dictated by the previous fixation type used and degree of bone resorption. In cases of massive osteolysis (tunnel diameter of ≥ 14 mm), the BTB autograft with bone plugs was used because it is larger as compared with ST/G autografts, while the ST/G autograft was employed only in cases with unexpanded tunnels. Of all patients who underwent ACL revision surgery, 43% received BTB autografts and 57% received ST/G autografts.

Surgical intervention was performed under spinal anesthesia; the patient was placed on a standard operating table. Standard anteromedial and anterolateral arthroscopy portals were used.

The surgical procedure was as follows. First, a graft choice was made. Then, the failed hardware (an old graft and fixation screws) was removed, and new tunnels were drilled in the femur and tibia using a standard technique. A new autograft was incorporated and fixed into the bone tunnels using the standard methods (i.e., Endobutton + interference screw fixation for ST/G autografts; Rigid-fix + interference screw fixation for BTB autografts). Graft tensioning was performed at full extension, as well as at 45° and 90° of flexion. A wound was closed in layers, with an active drainage system left in the joint cavity. All patients underwent thromboprophylaxis with enoxaparin sodim or dalteparin sodium.

### Postoperative regimen and recovery

Active drainage was removed 24 to 36 hours after surgery. A rigid knee brace was applied and maintained for 3 weeks. Knee flexion was only allowed at rehabilitation visits 8 days after surgery, whilst the full range of motion was allowed at the end of knee brace usage. For the first 3 weeks after the operation patients were prohibited from inflicting load on their operated lower limbs. 8 days after the operation patients were allowed partial loading of the operated limb (15–20 kg), which increased to half weight-bearing 15 days after the surgery. All patients were allowed full weight-bearing and to walk without crutches after 2 weeks of rehabilitation.

All patients in the comparison and experimental groups underwent a rehabilitation program, which included the use of a continuous passive motion machine, manual knee mobilization, lymphatic massage, electrical muscle stimulation, phonophoresis, and magnetic and laser therapy.

### Assessment

The outcomes were measured using the Lysholm knee questionnaire and the International Knee Documentation Committee (IKDC) questionnaire. Revision surgery patients were asked to fill in the forms before revision and postoperatively, at 1-year and 5-year follow-ups. A year after the operation, the survey embraced all patients in the study and control group. At 5 years after revision surgery, the survey involved only patients operated on before the first half of 2014 because other patients had incomplete follow-up at the time. Among them were 112 patients in the revision surgery group, 54 with hamstring autografts and 58 with BTB autografts, and 94 patients in the primary surgery group, 43 with hamstring autografts and 51 with BTB autografts.

Graft integrity after revision was assessed through MRI imaging at 6 months, at 1 year, and at 5 years postoperatively. For comparison purposes, a similar test was conducted in the control group.

### Data analysis

Data were processed in Excel 2016 (Microsoft Corp, Redmond, WA, USA) and STAT1ST1CA 10.0 (Statsoft, Tulsa, OK, USA). Spearman’s rank correlation and the Mann–Whitney U-test were used. The null hypothesis regarding the normality of distribution was rejected at p < 0.05.

### Ethics, funding, and potential conflicts of interest

The study was conducted in accordance with the ethical principles approved by the Ethics Committee of I.M. Sechenov First Moscow State Medical University (Protocol No. 4 of 22.03.2018) and in accordance with the World Medical Association Declaration of Helsinki. All the patients have given written informed consent. Tatyana Baltina was funded by the subsidy allocated to Kazan Federal University for the state assignment in the sphere of scientific activities, No 17.9783.2017/8.9. No conflicts of interest were declared.

## Results

### Bone tunnel positioning

1–2 bone tunnels were found to be malpositioned in 87/218 patients. Of these, 39% reported instability caused by injuries. The Spearman coefficient values indicated a relationship between instability and malposition of bone tunnels (р = 0.03).

### Baseline characteristics of study patients

In the study group, Lysholm scores demonstrated good knee function in 14 patients (6%; 77–86 points), fair in 64 patients (29%; 67–76 points), and poor in 140 patients (64%; < 66 points). The mean score was 44 (SD 11). According to the IKDC scores, 11 patients (5%; 80–89 points) have nearly normal knee function, 62 patients have abnormal function of the knee (28%; 70–79 points), and 145 patients have a very abnormal knee function (67%; < 70 points). The mean score was 39 (12).

### Outcomes at 1 year and 5 years after the revision surgery

Table 1 provides comparative surgical outcomes measured at 1 year and 5 years postoperatively.

The Mann–Whitney U-test shows statistically significant differences in the 1-year postoperative knee function between patients who underwent revision with BTB and hamstring autografts (p = 0.04). The differences were also statistically significant between the group of patients who had revision with hamstring autografts and the primary surgery patients (p = 0.04). There was no statistically significant difference between the patients who underwent revision with BTB autografts and the primary surgery patients. The 5-year outcomes show a similar trend.

Based on the results of the revision ACL surgery, BTB autografts performed better than ST/G autografts. The Lysholm and IKDC scores in patients who underwent revision with ST/G autografts did not improve significantly between the 1-year and 5-year follow-ups. The revision BTB group, on the other hand, demonstrated a statistically significant improvement (p = 0.04). In addition, the results of the revision ACL surgery with BTB autografts were found to be comparable to those after the primary ACL surgery. There were no statistically significant differences between patients who underwent 1-stage or 2-stage surgeries.

### Sports and recreation activity

The proportion of patients who did not participate in sports before injury did not change significantly but the proportion of professional players decreased in favor of amateur athletes, from 62% to 18% by the 5-year follow-up (Table 2). Thus, most patients after revision continued to play sports but a substantial number of them left professional sports and their level of sports participation changed. There were no statistically significant differences in sports participation between patients with BTB and ST/G autografts.

**Table 2.** Level of sports participation before and after revision

Sports participation level (%)	Before revision n = 218	After revision	
		1 year	5 years
		n = 218	n = 112
Not involved	23	25	29
Amateur	15	38	53
Professional	62	37	18

### Graft status

Graft survival at 1 and 5 years (p = 0.04 and p = 0.04, respectively) after revision is lower than after the primary operation (Table 3). However, a survival rate of 80% is rather high and consistent with the results of other studies. There were no statistically significant differences in survival between ST/G and BTB autografts (p = 0.09).

**Table 3.** Graft survival rates

Graft rupture (%)	Revision group			Primary group		
	6 months	1 year	5 years	6 months	1 year	5 years
	n = 218	n = 218	n = 112	n = 218	n = 218	n = 112
No	100	91	80	100	95	91
Partial	0	7	12	0	4	6
Complete	0	2	8	0	1	3

## Discussion

As the number of primary anterior cruciate ligament operations grows, revision surgery becomes an increasing area of interest. The ACL revision procedure depends on several factors: the position of bone tunnels, degree of bone resorption, previous graft type, and fixation choice.

The causes of graft failure include (Magnussen et al. 2012, Mariscalco et al. 2013):

1. preoperative: concomitant damage to the capsular-ligamentous knee apparatus (meniscus tear, cartilage defects);
2. intraoperative: improper graft choice, inadequate notch dimensions, improper positioning of bone tunnels, improper tensioning of graft, inadequate graft fixation;
3. postoperative: the lack of graft remodeling and revascularization, inadequate recovery program.

According to many researchers, the majority of recurrent instability episodes that emerge after ACL reconstruction relate to malpositioning of the femoral tunnel (Paterno et al. 2014, Yasuda et al. 2016, Ochi et al. 2017). Some studies even distinguish the incorrect position of bone tunnels as a main reason for the postoperative recurrence of instability, which requires revision in 70–80% of cases (Mariscalco et al. 2013). This is corroborated by our findings, as we noted malposition of the bone tunnels in 40% undergoing revision surgery.

Nowadays, there are 3 approaches to the creation of the femoral tunnel: transtibial technique, anteromedial technique, and retrograde technique. The latest studies (Rahardja et al. 2020), however, found no differences in the risk of revision between transtibial and anteromedial techniques at short-term follow-up. There was a slight difference in favor of the anteromedial portal technique detected a year after operation but the authors deemed it clinically insignificant, assuming that surgeons can achieve better results with any other method of tunnel creation (Rahardja et al. 2020).

Much has been written about the challenge of tunnel enlargement after primary ACL reconstruction by the use of an ST/G hamstring graft (Iorio et al. 2013, Zhang et al. 2014, Weber et al. 2015). Favored for a less traumatic influence on the donor area, ST/G hamstring grafts have experienced a large wave of popularity growth. There is biomechanical evidence showing that hamstring grafts are stronger that BTB grafts (Schimoler et al. 2015, Stolarz et al. 2016).

The widening of bone tunnels is driven by many factors such as the graft fixation technique, surgical approach, rehabilitation protocol, and the diversity of biological factors. The major challenge of using a hamstring tendon graft for ACL reconstruction is tendon–bone incorporation, as the biology of tendon graft–bone tunnel healing is incompletely understood (Chen 2009). Most authors agree that tunnel widening correlates indirectly with the clinical outcomes of the ACL reconstruction (Iorio et al. 2013, Weber et al. 2015). Yet, it poses a challenge during revision.

During this study, the BTB autografts proved to be more effective regarding long-term IKDC and Lysholm scores, as compared with ST/G hamstring autografts. The survival rate of revision grafts is 80%, which is lower than that of primary grafts. This result is consistent with previous studies (Grassi et al. 2017, Mohan et al. 2018).

We found no statistically significant differences in sports participation between patients having BTB and ST/G hamstring autografts. The return-to-sports rate following revision ACL reconstruction is lower than that after primary ACL surgery. A relatively high rate of return to sport at any level was reported in patients who underwent revision ACL reconstruction, but the rate of return to sport at pre-injury level was relatively low (Glogovac et al. 2019). We found that the majority of revision patients did not stop playing sports, but many switched from professional to amateur activities. This suggests that returning to the pre-injury level of physical activity after ACL revision may be a problem, but there is a good chance of retaining lower-level capabilities.

In conclusion, our results confirm a high degree of bone-tunnel malposition in patients undergoing revision ACL surgery due to recurrence of knee instability. BTB autografts outperformed ST/G autografts in the short and long term. The 5-year autograft survival after revision operation was around 80%, which was lower than that after primary intervention (∼91%). The postoperative level of sports participation decreased slightly.

**Table 1.** Postoperative knee function. Values are mean score (standard deviation)

Scale	1-year follow-up			5-year follow-up		
	Revision group		Primary group	Revision group		Primary group
	BTB	ST/G		BTB	ST/G	
IKDC	76 (5.4)	67 (9.1)	79 (7.3)	82 (9.2)	70 (11)	85 (7.7)
Lysholm	79 (7.7)	71 (11)	81 (6.7)	87 (10)	72 (12)	89 (8.5)

BTB = bone–patellar tendon–bone autografts,

ST/G = semitendinosus/gracilis hamstring autografts.
